# Supplementary material for: Elevated Expression and Activation of GPR15 in Immune Cells in Graves’ Disease
Source: Biomolecules. 2022 Dec 18;12(12):1899. doi: 10.3390/biom12121899 (PMC9776225; doi:10.3390/biom12121899)
Supplement: Supplementary file 1 [file biomolecules-12-01899-s001.zip › biomolecules-2031518-supplementary.pdf]

Supplementary Table S1. Demographics of participants.

Gene expression by qPCR	Graves' Disease	Health Control
Participants (female /total n)	(29/36)	(29/46)
Age(years)	34.0 ± 11.8	35.8 ± 10.2
**Flow cytometry**	**Graves' Disease**	**Health Control**
Participants (female /total n)	(24/29)	(19/28)
Age(years)	33.2 ± 10.4	33.6 ± 9.4
**ELISA of GPR15L**	**Graves' Disease**	**Health Control**
Participants (female /total n)	(28/34)	(21/33)
Age(years)	33.1 ± 10.3	34.7 ± 9.8
**Correlation analysis**	**Graves' Disease**	**Health Control**
Participants (female /total n)	(29/37)	-
Age(years)	33.1 ± 9.3	-

Supplementary Table S2. Primer sequences used in the study.

Gene	Forward	Reverse	Product Size
GPR15	GGGAGCTCACGCTGATTGAT	TGGTAATGGGCACACAGCTT	166
GPR15L	AGCCCCAACTCAACAAACCT	GTTCTCCGCTGTCTGGAGTC	145
IL-4	CCATGAGAAGGACACTCGCT	CGTACTCTGGTTGGCTTCCTT	151
IL-10	GTGAAAACAAGAGCAAGGCCG	TCGCCACCCTGATGTCTCA	159
IL-17a	CAATCCCACGAAATCCAGGATG	GGTGGAGATTCCAAGGTGAGG	156
IL-21	AAGCTCCCAAGGTCAAGATCG	AGCAGGAAAAAGCTGACCACT	155
IL-22	GTGCAAAAGCTGAAGGACACA	TTCTAGCAGGGAAAGGGGGT	157
IFN-γ	AGGCTTTATCTCAGGGGCCA	AGCACTGGCTCAGATTGCAG	157
β-actin	CATTGCCGACAGGATGCAG	CTCGTCATACTCCTGCTTGCTG	169

Supplementary Table S3. Antibodies used for flow cytometry.

Flow cytometry of PBMCs	Flourochrome	Clone	Provider
Fixable Viability Dye	eFluor 780	-	invitrogen
CD3	BUV395	SK7	BD
CD4	Brilliant Violet 605	RPA-T4	BioLegend
CD8	PE-Cyanine7	RPA-T8	invitrogen
CD19	eFluor 506	HIB19	invitrogen
CD14	Brilliant Violet 510	M5E2	BioLegend
CD16	eFluor 450	eBioCB16	invitrogen
CD56	eF450	TULY56	invitrogen
CD25	APC	BC96	invitrogen
CD127	PE-Cyanine7	eBioRDR5	invitrogen
CD45RA	eFluor 450	HI100	invitrogen
CCR7	APC	3D12	invitrogen
GPR15	PE	367902	R&D systems

Supplementary Table S4. Clinical characteristics of IHC subjects in this study.

	Graves' Disease	Health Control	P value
Participants (female /total)	5/5	4/6	0.46
Age (years)	34.2±7.4	51.8±11.3	<0.05
FT3 (pmol/L)	3.33(3.32,3.35)	4.51(4.38,4.89)	<0.01
FT4 (pmol/L)	11.18(6.11,12.30)	16.26(14.92,16.68)	<0.01
TSH (uIU/ml)	7.19(4.62,9.77)	2.43(1.58,2.51)	<0.05
TrAb (IU/L)	7.83(4.03,11.86)	0.58(0.45,0.83)	0.06
TPOAb (IU/ml)	93.37(45.40, 137.70)	15.07(11.77, 18.28)	0.06

IHC: Immunohistochemistry

Supplementary Figure S1

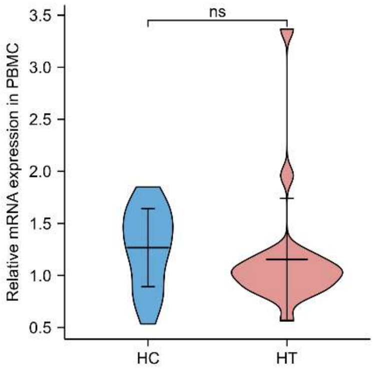

Figure S1. GPR15 mRNA expression was not change in Hashimoto's thyroiditis patients. HC: health controls, HT: Hashimoto's thyroiditis. (ns, p⩾0.05)

Supplementary Figure S2

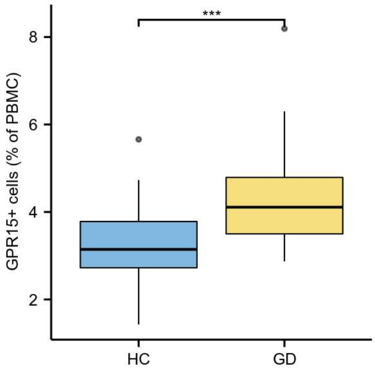

Figure S2. Frequency of GPR15+ cells among PBMCs in both groups. GPR15+: GPR15-expressing; HC: health controls, GD: Graves' disease. (***, p<0.001)

Supplementary Figure S3

Characteristics(frequency,%)	OR (95% CI)	*P* value
Total GPR15+	1.248(1.019−1.581)	<0.05
B cells GPR15+	0.933(0.791−1.090)	0.16
T cells GPR15+	2.543(1.373−5.505)	<0.01
Th GPR15+	1.491(1.034−2.287)	<0.05
Tc GPR15+	1.556(0.873−2.937)	0.06
Treg GPR15+	1.146(0.824−1.624)	0.23
NK cells GPR15+	8.066(1.733−57.250)	<0.01
Monocytes GPR15+	2.843(1.516−7.069)	<0.001

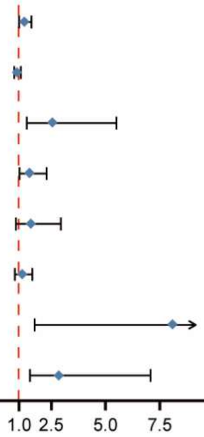

Figure S3. Odds ratios (OR) of the associations of GPR15+ cells frequency in PBMCs with GD after adjusting for age and gender.
